# Sour Finding on Popular Sweetener: Increased Cancer Incidence Associated with Low-Dose Aspartame Intake

**DOI:** 10.1289/ehp.114-a176a

**Published:** 2006-03

**Authors:** M. Nathaniel Mead

More than 20 years have elapsed since aspartame was approved by regulatory agencies as an artificial sweetener. But scientists draw conclusions on carcinogenicity based on the evidence available at the time, and new research out of the European Ramazzini Foundation of Oncology and Environmental Sciences bolsters recent calls for reconsideration of regulations governing aspartame’s widespread use in order to better protect public health, particularly that of children **[*EHP*** **114:379–385; Soffritti et al.]**.

The researchers added aspartame to the standard diet of Sprague-Dawley rats, using dosages designed to simulate a wide range of human intakes. Each rat was observed from 8 weeks of age until death. This is in contrast with earlier studies that typically sacrificed animals between 104 and 110 weeks of age, corresponding to about two-thirds of a rat’s lifespan (in humans, approximately 80% of cancer diagnoses are made in the last third of life, after age 55). Deceased animals were examined for microscopic changes in various organs and tissues, enabling a comprehensive assessment of aspartame’s carcinogenic potential. A total of 1,800 rodents were included, far more than in previous studies.

Aspartame-fed females showed significant evidence of lymphomas/leukemias and of carcinomas of the renal pelvis and ureter. The effect on the renal pelvis was much more evident when carcinomas were combined with atypical preneoplastic lesions. The researchers also observed an insignificant increase in incidence of malignant schwannomas of the peripheral nerves in males, as well as hyperplasia of the olfactory epithelium in males and females. Lesions of the kidney and olfactory epithelium are extremely rare in this strain of rats and therefore merit special attention.

The carcinogenic effects were evident at daily doses as low as 400 parts per million, equivalent to an assumed daily human intake of 20 milligrams per kilogram body weight (mg/kg). This dosage is much less than the acceptable daily intake for humans, with current limits set at 50 mg/kg in the United States and 40 mg/kg in Europe. Surveys of aspartame intake in the United States and Europe from 1984 to 1992 showed that consumers typically consumed 2–3 mg/kg daily, with small children and women of child-bearing age consuming slightly more, at 2–5 mg/kg daily.

The public health implications of these findings may be substantial, since aspartame is used in about 6,000 products, and more than 200 million people regularly consume aspartame through foods, beverages, drugs (such as chewable vitamins), and hygiene products (such as toothpaste). Because the study did not take into account *in utero* and perinatal exposures, the authors point to this as a salient direction for future research, given that children and pregnant and breastfeeding women are among the major consumers of aspartame.

## Figures and Tables

**Figure:**
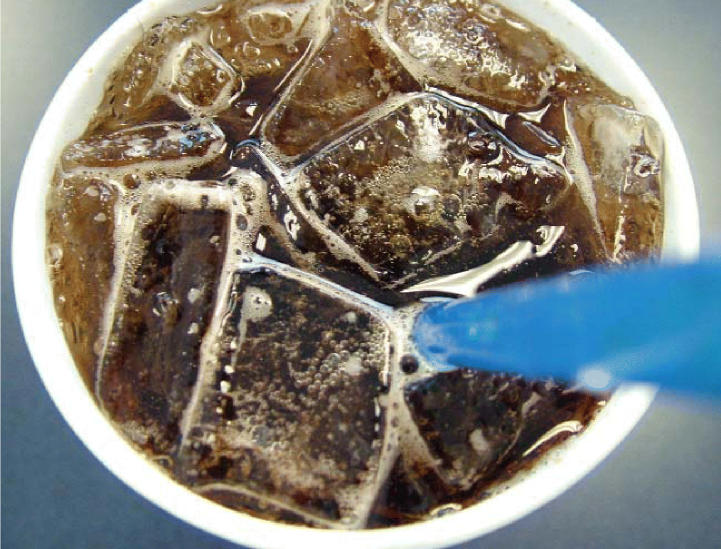
Stirring debate. New rodent data on aspartame, an artificial sweetener used in a variety of consumer goods, suggest the chemical’s potential cancer effects deserve more study.

